# Seroprevalence of SARS-CoV-2 immunoglobulin G in HIV-positive and HIV-negative individuals in KwaZulu-Natal, South Africa

**DOI:** 10.4102/ajlm.v12i1.2065

**Published:** 2023-06-29

**Authors:** Kerri-Lee A. Francois, Nokukhanya Msomi, Kerusha Govender, Lilishia Gounder, Pravi Moodley, Raveen Parboosing, Indrani Chetty, Lunga Xaba, Aabida Khan

**Affiliations:** 1 Discipline of Virology, Faculty of Health Sciences, School of Laboratory Medicine and Medical Sciences, College of Health Sciences, University of KwaZulu-Natal, Durban, South Africa; 2 National Health Laboratory Services, Inkosi Albert Luthuli Central Hospital, Durban, South Africa; 3 Department of Medical Virology, Faculty of Health Sciences, University of the Witwatersrand, Johannesburg, South Africa; 4 Discipline of Virology and National Health Laboratory Service, Inkosi Albert Luthuli Central Hospital, Durban, South Africa

**Keywords:** SARS-CoV-2 IgG prevalence, seroprevalence HIV-positive and HIV-negative, anti-SARS-CoV-2 IgG HIV-positive and negative, seroprevalence SARS-CoV-2 IgG, KwaZulu-Natal

## Abstract

**Background:**

KwaZulu-Natal ranked second highest among South African provinces for the number of laboratory-confirmed cases during the second wave of the severe acute respiratory syndrome coronavirus 2 (SARS-CoV-2) pandemic. The seroprevalence of SARS-CoV-2 among certain vulnerable groups, such as people living with HIV in KwaZulu-Natal, is unknown.

**Objective:**

The study aimed to determine the prevalence of SARS-CoV-2 immunoglobulin G (IgG) in HIV-positive versus HIV-negative patients.

**Methods:**

This was a retrospective analysis of residual clinical blood specimens unrelated to coronavirus disease 2019 (COVID-19) submitted for diagnostic testing at Inkosi Albert Luthuli Central Hospital, Durban, from 10 November 2020 to 09 February 2021. Specimens were tested for SARS-CoV-2 immunoglobulin G on the Abbott Architect analyser.

**Results:**

A total of 1977/8829 (22.4%) specimens were positive for SARS-CoV-2 antibodies. Seroprevalence varied between health districts from 16.4% to 37.3%, and was 19% in HIV-positive and 35.3% in HIV-negative specimens. Seroprevalence was higher among female patients (23.6% vs 19.8%; *p* < 0.0001) and increased with increasing age, with a statistically significant difference between the farthest age groups (< 10 years and > 79 years; *p* < 0.0001). The seroprevalence increased from 17% on 10 November 2020 to 43% on 09 February 2021 during the second wave.

**Conclusion:**

Our results highlight that during the second COVID-19 wave in KwaZulu-Natal a large proportion of people living with HIV were still immunologically susceptible. The reduced seropositivity in people with virological failure further emphasises the importance of targeted vaccination and vaccine response monitoring in these individuals.

**What the study adds:**

This study contributes to data on SARS-CoV-2 seroprevalence before and during the second wave in KwaZulu-Natal, South Africa, which has the highest HIV prevalence globally. Reduced seropositivity was found among people living with HIV with virological failure, highlighting the importance of targeted booster vaccination and vaccine response monitoring.

## Introduction

Coronavirus disease 2019 (COVID-19), caused by severe acute respiratory syndrome coronavirus 2 (SARS-CoV-2), is one of the most significant pandemics in history. In Africa, South Africa has the highest number of confirmed cases (Africa CDC COVID-19 Dashboard, 2021 – https://africacdc.org/covid-19/). KwaZulu-Natal province ranked second-highest nationally for the number of laboratory-confirmed cases during the second wave of the SARS-CoV-2 epidemic in South Africa.^1^

The first wave in KwaZulu-Natal was around June 2020 – July 2020. The wave was followed by lower but sustained viral circulation between August 2020 and October 2020 and a more explosive second wave from November 2020 to February 2021. The second wave was of greater magnitude than the first, with the emergence of the more transmissible beta variant.^2,3^ The third wave of the epidemic in South Africa started in May 2021, with a resurgence in KwaZulu-Natal in June 2021. The more transmissible delta variant rapidly displaced the beta variant between May 2021 and August 2021, while the even more highly transmissible omicron variant overtook the delta variant between November 2021 and January 2022.^2,4^

Polymerase chain reaction (PCR) is most accurate for the diagnosis of acute SARS-CoV-2 infection.^5^ However, the use of PCR-confirmed cases for reporting may underestimate the true extent of the SARS-CoV-2 pandemic. Although not indicated for the diagnosis of acute infection, serology may give a more accurate reflection of the true prevalence of infection in people who may not have had a PCR test during acute infection or who were asymptomatic.^6^

Even though South Africa has passed the third, fourth and fifth waves of the SARS-CoV-2 epidemic and is currently experiencing a transition phase, serological surveillance remains a vital tool to understand the true extent of SARS-CoV-2 exposure and inform public health responses.^7^

Seroprevalence in 2020 was estimated to be 5–10 times higher than reported cases identified by PCR.^8,9,10^ A population-based seroepidemiological survey in Gauteng province in South Africa, that started 8 weeks into the first wave and ended at the peak of the second wave, estimated that there were 2.89 million SARS-CoV-2 infections, which is 7–8 times higher than the reported 332 000 PCR cases.^10^ Local SARS-CoV-2 seroprevalence rates were over 60% in black South African blood donors^11^ and 30% – 40% among patients accessing care during the first wave in the Cape Town Metropolitan sub-districts.^12^ Similarly, seroprevalence studies in Spain, Geneva and New York reported seroprevalence higher than the number of reported cases.^13,14,15^

Ideally, population-based serosurveys should be used to estimate seroprevalence nationally.^16^ However, serosurveys require extensive resources, and involve active recruitment and prospective follow-up of individuals. Using residual clinical specimens submitted for either routine screening or diagnostic testing could provide a snapshot^16,17^ and is a convenient way to conduct passive surveillance. In South Africa, seroprevalence studies using convenience specimens have been conducted by the South African Blood Bank Services (SANBS) in the Western Cape and Gauteng.^11,12,18^

KwaZulu-Natal has the world’s highest HIV prevalence^19^ and has the second highest number of reported SARS-CoV-2 infections nationally. Second-wave infections in South Africa caused by the beta variant were associated with increased disease severity, especially in people living with HIV^20^ when no vaccines were available. Thus, an accurate estimate of SARS-COV-2 seroprevalence among people living with HIV was important in the early phase of the SARS-CoV-2 epidemic for outbreak surveillance, implementation of vaccination strategies, and to assess the effectiveness of non-pharmaceutical interventions in KwaZulu-Natal. We therefore determined the SARS-CoV-2 immunoglobulin G (IgG) seroprevalence among people with HIV infection in KwaZulu-Natal.

## Methods

### Ethical considerations

This study was approved by University of KwaZulu-Natal Biomedical Research Ethics Committee (Ref. BCA256/010). Informed patient consent was not required as this was a retrospective study using residual diagnostic specimens. No additional specimens were collected. All residual clinical specimens were de-identified and labelled with a unique study number; patient privacy and confidentiality data were protected in accordance with the Declaration of Helsinki. Data were collected in a password-protected Excel spreadsheet (Microsoft Corp., Redmond, Washington, United States) on a dedicated computer behind firewall-protected servers, which was only accessible to the primary investigator.

### Study design

The authors retrospectively tested residual clinical sera and plasma specimens submitted to the Department of Virology at Inkosi Albert Luthuli Central Hospital, Durban, for diagnostic testing unrelated to COVID-19 from 10 November 2020 to 09 February 2021, representing the period before and during the second wave (22 November 2020 to 27 March 2021) of the SARS-CoV-2 epidemic in KwaZulu-Natal.

### Sample size

A minimum sample size of 7294 was estimated using OpenEpi (Version 3.1; http://www.openepi.com/)^21^ based on a SARS-CoV-2 prevalence of 5% to 10% before the second wave peak with a maximum 1% margin error and using published seroprevalence data from studies done globally (5%,^10^ 1% – 6.9%^22^ and 1% – 10%^8^), the assumed point prevalence was calculated.

Using the STATA^®^ (StataCorp, College Station, Texas, United Staes) sampsi function, the sample size was calculated to compare SARS-CoV-2 seropositivity in HIV-positive and HIV-negative individuals with a power of 80%, two-sided alpha of 0.05 and equal size in each group. A sample size of 3682 would be sufficient to detect a 1% difference if the prevalence is low (1%) and a 3% difference if the prevalence is high (13%).^8,13,22^

### Specimen selection

Sera and plasma specimens with sufficient volumes from the viral serology (HIV and hepatitis B enzyme-linked immunosorbent assay and HIV viral load laboratory sections) were selected for SARS-CoV-2 IgG testing. Specimens with inadequate volumes (< 100 µL) were excluded. The selected specimen results were linked to demographic information extracted from the laboratory information system. Specimens from the same patient were tested once only.

### Laboratory methods

Testing for SARS-CoV-2 IgG was performed on the Abbott Architect using the Abbott Architect SARS-CoV-2 IgG assay (Abbott Diagnostics, Abbott Park, Illinois, United States).^23^ Sera and plasma specimens were stored at 2 °C – 8 °C for not more than 7 days and −20 °C for long-term (> 7 days) storage. The assay is an automated two-step chemiluminescent microparticle immunoassay for qualitative detection of SARS-CoV-2 IgG antibodies against the SARS-CoV-2 nucleocapsid protein. The assay was performed and interpreted according to the manufacturer’s instructions.^24^

### Statistical analysis

Statistical analysis was done using STATA^®^ version 15 (StataCorp, College Station, Texas, United States). We performed a logistic regression analysis to determine whether age, gender, HIV seropositivity, and HIV viral load were associated with SARS-CoV-2 seropositivity, with significance level set to 0.05.

## Results

A total of 9083 specimens were tested. However, 254 specimens were excluded from the analysis due to existing SARS-CoV-2 IgG test requests (*n* = 248) or mismatched laboratory information (*n* = 6). A total of 8829 (Table 1) specimen results were analysed: 5724 (64.8%) of the specimens were from female and 2865 (32.5 %) from male patients, while 240 (2.7%) had no specified gender; 1309 (14.8%) of sera specimens had HIV enzyme-linked immunosorbent assay results, and 5522 (62.5%) of plasma specimens had HIV viral load results.

**TABLE 1:** Demographic characteristics of specimens collected in KwaZulu-Natal, South Africa, from 10 November 2020 to 09 February 2021.

Category	Total	IgG positive	IgG negative	% Seropositivity	Odds ratio	95% CI	*p*
**Total per category**	8734	1955	6779	22.4	-	-	-
**Age categories in years**	-	-	-	-	1.012*	1.008–1.017*	< 0.0001
< 10 years	390	39	351	10.0	-	-	-
10–19 years	601	97	504	16.1	-	-	-
20–29 years	1982	451	1531	22.8	-	-	-
30–39 years	2677	603	2074	22.5	-	-	-
40–49 years	1665	363	1302	21.8	-	-	-
50–59 years	893	241	652	27.0	-	-	-
60–69 years	364	109	255	29.9	-	-	-
70–79 years	135	47	88	34.8	-	-	-
> 79 years	27	5	22	18.5	-	-	-
Age unknown	95	22	73	23.2	-	-	-
**Gender**	-	-	-	-	-	-	< 0.0001
Female	5724	1350	4374	23.6	Reference	-	-
Male	2865	566	2299	19.8	0.705	0.617–0.806	-
Unknown	240	61	179	23.6	-	-	-
**HIV seropositivity**	-	-	-	-	-	-	< 0.0001
HIV-negative	1260	445	815	35.3	Reference	-	-
HIV-positive	5571	1058	4513	19.0	0.402	0.349–0.463	-
HIV status unknown	1998	474	1524	23.7	-	-	-
**HIV viral load (copies/mL)**	-	-	-	-	-	-	< 0.0001
< 1000	4809	977	3832	20.3	Reference	-	-
> 1000	713	75	638	10.5	0.518	0.401–0.669	-

CI, confidence interval; IgG, immunoglobulin G.

* , Seropositivity increased with increasing age, with a 1.012 increase in odds for every year increase in age (*p* < 0.0001).

**FIGURE 1:**
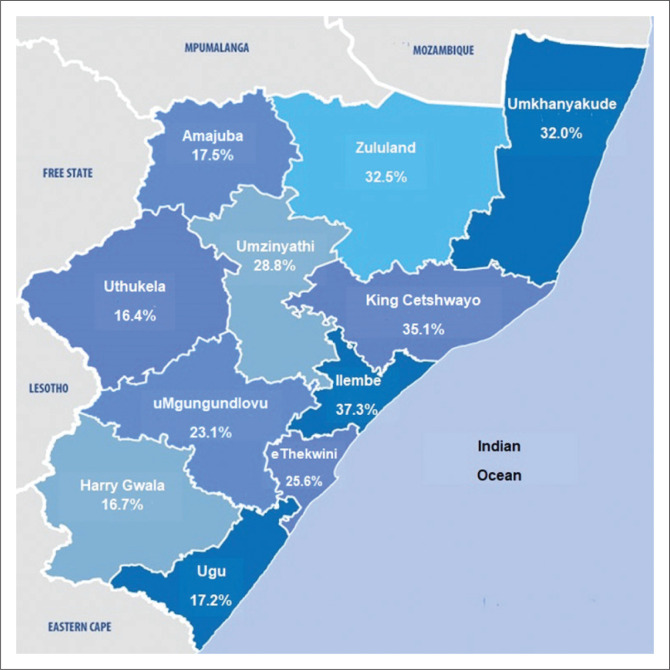
SARS-CoV-2 immunoglobulin G seropositivity per district in KwaZulu-Natal, South Africa, from 10 November 2020 to 09 February 2021.

**FIGURE 2:**
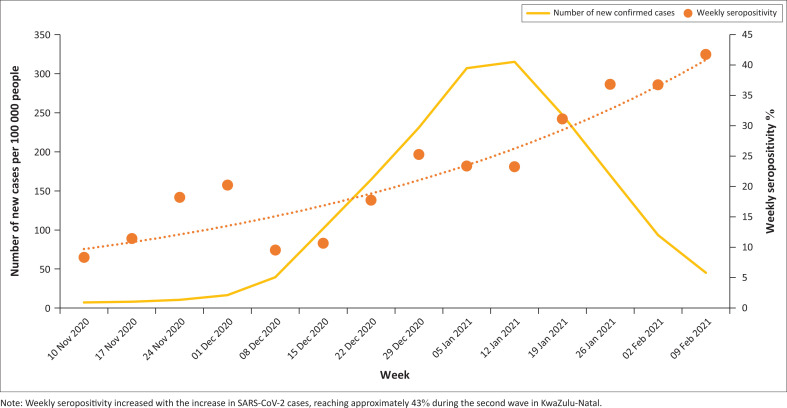
Temporal relationship between seropositivity and polymerase chain reaction positivity of specimens collected in KwaZulu-Natal, South Africa, from 10 November 2020 to 09 February 2021.

The average SARS-CoV-2 seropositivity across 11 health districts (Figure 1) during the second wave was 22.4%; the iLembe district had the highest seropositivity (37.3%), followed by King Cetshwayo (35.1%), and uMkhanyakude (32%). Weekly seropositivity increased with increasing SARS-CoV-2 cases (Figure 2), reaching 43% during the second wave in KwaZulu-Natal.

**FIGURE 3:**
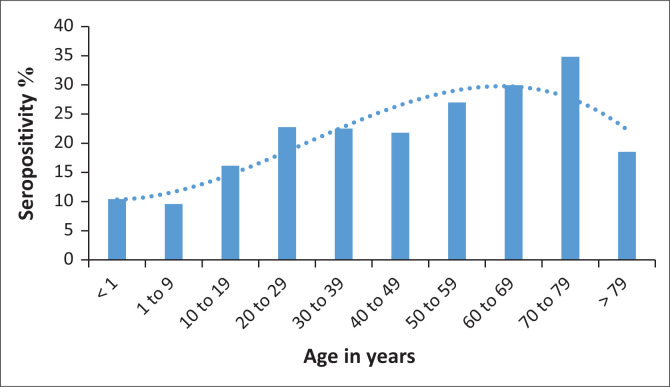
Age and seropositivity of specimens collected in KwaZulu-Natal, South Africa, from 10 November 2020 to 09 February 2021.

Seropositivity among female patients was 23.6% (odds ratio: 0.705, 95% confidence interval: 0.617–0.806) while male patients had lower odds of seropositivity (*p* < 0.0001) (Table 1). Seropositivity increased with increasing age, with a 1.012 increase in odds for every year increase in age (*p* < 0.0001) (Figure 3). Seropositivity was highest in the 70–79 year age group and lowest in those under 9 years of age. Seropositivity (Table 1) was higher in the HIV-negative compared to the HIV-positive (35.3% vs 19%; odds ratio: 0.402; 95% confidence interval: 0.349–0.463). HIV positivity with a viral load greater than 1000 copies/mL was associated with lower odds of SARS-CoV-2 seropositivity (odds ratio: 0.518; 95% confidence interval: 0.401–0.669). No statistically significant interaction (at a 10% level) was noted between HIV status and age or gender.

## Discussion

We assessed the SARS-CoV-2 IgG seropositivity in convenience specimens submitted to the Department of Virology at Inkosi Albert Luthuli Central Hospital, Durban, before and during the peak of the second COVID-19 wave caused by the beta variant. We observed an increase in seropositivity from 17% before to 43% during the second wave in KwaZulu-Natal, reflective of the increase in the number of infected individuals during the second wave, with a mean SARS-CoV-2 seropositivity of 22.4% across the study period. There were statistically significant associations between SARS-CoV-2 seropositivity, age, gender, and HIV status. Our results indicate that the true extent of the SARS-CoV-2 epidemic in KwaZulu-Natal may have been underestimated due to reporting of PCR results alone. This is similar to other local seroprevalence studies that report higher seroprevalence compared to the number of reported PCR cases.^10,11,12,25^

The seropositivity in our study was lower than the seroprevalence reported by surveillance studies in Cape Town (40%) and Gauteng (27.8%).^12,18^ The lower prevalence may be because the specimens were not collected immediately after the first wave in KwaZulu-Natal; they were collected 4 months later, thus affecting the detection of IgG, which wanes over time. The sentinel surveillance studies were started directly after the first wave in both Cape Town and Gauteng.

Furthermore, the majority (63%) of the specimens in our study were from HIV-positive individuals; 10% of these specimens had an HIV viral load greater than 1000 copies/mL and reduced SARS-CoV-2 seropositivity. HIV viral load greater than 1000 copies/mL had a statistically significant association with lower odds of SARS-CoV-2 IgG positivity. HIV infection is associated with a reduced immune response to infectious pathogens.^26^ However, in individuals on antiretroviral therapy, immune responses to SARS-CoV-2 are reported to be similar to that in HIV-negative individuals.^27,28^ People on antiretroviral therapy may display immune reconstitution;^26^ however, chronic immune activation and impaired B-cell responses in individuals on antiretroviral therapy with virologic failure may result in reduced antibody responses^26,28^ and lower IgG concentrations,^29^ accounting for the lower seropositivity in those with HIV infection.

Lower SARS-CoV-2 IgG concentrations may affect the detectability of IgG,^30^ which may be lower than the detectable limit of the assay. Based on a local evaluation of the performance of the Abbott Architect SARS-CoV-2 IgG assay, sensitivity is highest 30–41 days after a positive PCR, following moderate to severe infection.^23^ However in our study reduced seropositivity was most likely due to reduced immunity in people with uncontrolled HIV and the timing of sampling in relation to the epidemic waves.^23^

We have demonstrated that SARS-CoV-2 seropositivity increased with age, with children under 10 years having the lowest seropositivity (11.1%). Similar patterns have been described locally^25^ and internationally.^15^

Our reported seropositivity (10%) in children under 10 years during the second wave in KwaZulu-Natal indicated that these children may have been more vulnerable to SARS-CoV-2 infection. However, the rate of infections in South Africa among these children was 1.4%,^31^ suggesting that PCR testing alone may have underestimated infection rates or that these children were asymptomatic. However, a seroprevalence in children under 9 years during the first wave in Gauteng was reported to be about 20%,^18^ most likely due to Gauteng having the highest infection rate nationally during the entire epidemic.^1^

We found no discernible pattern in the district seroprevalence during the second wave. However, the rate of infections in the Ethekwini, iLembe, Ugu, and Uthukela districts were subsequently lower in the third wave.^1^

### Limitations

Firstly, the specimens tested were from individuals accessing public healthcare and thus may not represent the general population. However, it potentially represents people living with HIV accessing antiretroviral therapy in our province.

Secondly, surveillance using residual clinical specimens without exposure time data, as done in this study, may underestimate seropositivity.^32^ Thirdly, seropositivity may be underestimated since SARS-CoV-2 IgG detection and durability is directly proportional to disease severity.^33,34^ Although antibody positivity was still high in another study at 8 months after mild and asymptomatic infection,^35^ overall detectability and durability may be reduced in HIV-positive persons.^36^

Fourthly, the Abbott Architect SARS-CoV-2 IgG assay detects IgG to nucleocapsid antigens only and at the time was the only approved commercial serological assay by the South African Health Products Regulatory Authority.^37^

The use of nucleocapsid IgG solely to determine seroprevalence may underestimate the true rate of past infections.^32^ Nucleocapsid IgG declines more rapidly than spike IgG. However, nucleocapsid IgG is more sensitive within the first 14 days of SARS-CoV-2 infection as compared to spike IgG.^34^ Lastly, antibody cross-reactivity with other Betacoronaviruses may lead to false positives.^23,38^ However, the Abbott Architect SARS-CoV-2 IgG has a specificity of 99%.^23,38^

### Conclusion

Our results highlight that after the second wave in KwaZulu-Natal, many people accessing public healthcare were immunologically susceptible. Furthermore, reduced seropositivity in HIV-positive individuals with HIV viral loads greater than 1000 copies/mL further emphasised the importance of targeted vaccination of people living with HIV and monitoring response to vaccination in these individuals.^39,40^
